# Over Ten-Year Kidney Graft Survival Determinants

**DOI:** 10.1155/2012/302974

**Published:** 2012-11-19

**Authors:** Anabela Malho Guedes, Jorge Malheiro, Isabel Fonseca, La Salete Martins, Sofia Pedroso, Manuela Almeida, Leonídio Dias, António Castro Henriques, António Cabrita

**Affiliations:** ^1^Serviço de Nefrologia, Hospital de Santo António, 4099-001 Porto, Portugal; ^2^Serviço de Nefrologia, Hospital de Faro, Rua Leão Penedo, 8000 Faro, Portugal

## Abstract

Kidney graft survival has been mainly evaluated using an up to 10-year threshold. Instead, in this study our aim was to evaluate predictive variables that impact long-term kidney graft survival (≥10 years). We enrolled 892 patients in our analysis: 638 patients with functioning graft at 10 years PT and 254 patients with graft failure at 10 years PT (considering patient death with a functioning graft <10 years PT as graft failure). Between groups comparisons were done using Mann-Whitney and chi-square test. To determine independent predictive variables for long-term graft survival a multivariate-adjusted logistic regression was performed. Significant predictors of long term graft survival were lower 12-month PT creatinine (OR = 0.26, *P* < 0.001), lower donor age (OR = 0.98, *P* = 0.004), shorter time on dialysis (OR = 0.93, *P* = 0.044), recipient positive CMV IgG (OR = 1.59, *P* = 0.040), absence of AR episodes (OR = 1.57, *P* = 0.047), 0 to 1 (versus 2) HLA-B mismatch (OR = 1.80, *P* = 0.004), and recipients male gender (OR = 1.84, *P* = 0.005). Our results show that an early KT, younger donor age, and an optimal first year graft function are of paramount importance for long-term graft survival. Measures that address these issues (careful donor selection, preemptive KT, and effective immunosuppressive protocols) are still warranted.

## 1. Introduction

For patients with end-stage renal disease (ESRD), a successful transplant (compared with dialysis) provides significantly higher survival rates and better quality of life [1–3], which is directly linked to maintained function of the graft [4]. Since the 1980s, significant progress has been made in graft and patient survival rates after kidney transplant (KT) [5, 6]. As short-term results have improved, more patients with ESRD are opting for a transplant and more are surviving with functioning grafts into the second and third decades posttransplant [4, 5]. However, the vast majority of transplant recipients still experience progressive kidney graft dysfunction, ultimately leading to the development of graft failure [7]. Therefore, improving the long-term survival of KT by identifying modifiable risk factors is an important subject in the field of organ transplantation. Most research on KT outcomes has been focused on the first decade posttransplant (PT). The aim of our study was to analyze the characteristics and other variables that impact long-term kidney graft survival (beyond 10 years after KT).

## 2. Methods

### 2.1. Clinical Data

We conducted a retrospective study and analyzed data from a total of 996 kidney transplants (KT) performed between July of 1983 and June of 2000, at Hospital de Santo António, Porto, Portugal. After discharge, recipients were followed at our outpatient clinic until graft loss or death. Recipient and donor information was collected from our computed database. 

We included, in our analysis, all recipients with a graft survival beyond 3 months, and patients surviving beyond the first-year PT. Simultaneous multiple grafts recipients were excluded. Graft loss was defined as return to chronic dialysis, graft removal, a retransplant, or death.

We enrolled 892 patients in our analysis: 638 patients with functioning graft at 10 years of followup (Group I) and 254 patients with graft failure within 10 years PT (considering patient death with a functioning graft <10 years PT as graft failure) (Group II). 

Delayed graft function was defined as the need for one or more dialysis treatments in the first posttransplant week. 

### 2.2. Statistical Analysis

All statistical analysis was performed using SPSS software for Windows (version 18.0; Chicago, USA). Results are presented as median and interquartiles range for continuous variables and as frequency and percentages (*n*, %) for categorical variables. *P*  values < 0.05 were considered statistically significant. For comparison between groups the Mann-Whitney test and chi-square test were used for continuous nonparametric variables and categorical variables, respectively. To determine independent predictive variables for long-term graft survival a multivariate-adjusted logistic regression was performed, adjusting for age and sex of the recipient, mean time on dialysis, recipient status for cytomegalovirus IgG, prior blood transfusions (<3 versus ≥3), donor's age, number of HLA-B mismatches (0, 1 versus 2), PRA (<30 versus ≥30%), induction with antithymoglobulin, delayed graft function, acute rejection, and serum creatinine at 12 months after KT.

## 3. Results

### 3.1. Global Population

The majority of the enrolled patients were men (59.4%), with median age 37.0 years [IQR 27.6–47.1] at the time of transplant. Median time on dialysis was 2.6 years [IQR 1.4–5.0], 96.3% were on hemodialysis. Most donors were also men (70.9%). Median posttransplant follow-up time was 176.2 months [IQR 138.2–222.4]. The median survival time of renal grafts was 149.9 months [IQR 109.6–190.8]. Enrolled population characteristics are presented in Table 1.

### 3.2. Characteristics Comparison between KT Groups: Graft Function after Ten Years versus Graft Failure within Ten Years

The group with graft function after ten years (Group I-G I) had more frequently positive status for cytomegalovirus IgG (73.9% versus 62.1%, *P* = 0.001) and fewer pretransplant blood transfusions (cutoff <3 versus ≥3 units; 36.8% versus 45.3%, *P* = 0.02). As for donor characteristics both groups had similar gender distribution and serum creatinine by the time of death, but the group with longer graft function had younger donors (24.0 versus 29.0, *P* < 0.001) (Table 2). 

Group I being more frequently received to the induction therapy with ATG (45.5% versus 39.0%, *P* = 0.005) had less human lymphocyte antigen B (HLA-B) mismatches (*χ* ^2^ for trend, 2 HLA-B mismatches: 46.3% versus 58.7%, *P* = 0.002), lower PRA level (3.5% versus 7.6%, *P* = 0.005), and lower incidence of delayed graft function (29.3% versus 42.1%, *P* = 0.001), and episodes of acute rejection (25.3% versus 47.2%, *P* < 0.001) were less frequent. Serum creatinine at 12 months after KT was also lower in this group (1.30 versus 1.60, *P* < 0.001).

### 3.3. Long-Term Graft Survival Predictors

The multivariate analysis (Table 3) showed as significant predictors for better graft survival beyond 10 years lower 12-month posttransplant creatinine (OR = 0.26, *P* < 0.001), lower donor age (OR = 0.98, *P* = 0.004), shorter time on dialysis (OR = 0.93, *P* = 0.044), recipient positivity for CMV IgG (OR = 1.59, *P* = 0.040), absence of acute rejection episodes (OR = 1.57, *P* = 0.047), 0 to 1 (versus 2) HLA-B mismatch (OR = 1.80, *P* = 0.004), and recipient male gender (OR = 1.84, *P* = 0.005). Recipient age at transplant, number of transfusions (<3 versus ≥3), PRA level (<30 versus ≥30%), immediate graft function and induction with ATG were not significant predictors.

## 4. Discussion

This study focused only on patients with more than 10 years of followup, referring to kidney transplant performed between the year of 1983 and 2000. Major differences of this cohort of patients from kidney transplantation nowadays consist of different and less effective immunosuppression, initially based on azathioprine and prednisolone, then based on cyclosporine and evolving towards introduction of mycophenolate mofetil and tacrolimus. Infectious prophylaxis was also less effective, translating to higher incidence of CMV disease. Due to improvements related with these issues significant progress has been made in graft and patient survival rates after kidney transplant, since the 1980s [5, 6]. The half-life for deceased and living related allografts have improved to 13.8 and 21.6 years, respectively [8]. Our analysis showed a graft survival rate of 71.5% after ten years of follow-up. This increase has been mostly attributed to improvements in first-year survival [9].

When this population was submitted to kidney transplantation expanded criteria donors were not included, and the vast majority were deceased donors (99%), but many other factors are common to most survival analyses in renal transplantation, such as donor age or gender and creatinine level at 12 months. In this study, univariate analysis and multivariate logistic model were used for analyzing factors influencing renal graft survival. Data analysis indicated that an optimal first year graft function (including absence of acute rejection episodes and creatinine level at 12 months) was of paramount importance for long-term graft survival.

Several studies implicated acute rejection as a major risk factor for chronic allograft failure [10–12]. A previous report showed that an episode of acute rejection reduced from 12.5 to 6.6 years the kidney graft half-life [7]. Moreover, after an acute rejection, the development of a chronic rejection becomes more likely. In particular, early acute rejection, frequent acute rejection episodes, and refractory acute rejection can drastically affect long-term survival of a renal graft [7, 13, 14]. The data we presented in this study show that the incidence of acute rejection in the long-term survival patient group was significantly lower than that of the control group and a predictor of graft survival. In our population this could be explained by the use of less effective immunosuppression, reporting to the state of the art before the year 2000. In our study, any rejection episodes occurring later than one year posttransplantation were not analyzed.

The correlation between the elevated serum creatinine levels at 12 months and graft survival has been documented in several studies [11, 15]. In our study a lower 12-month PT creatinine was a strong determinant for long term kidney graft survival. 

Other independent known determinants for graft survival include recipient age at transplantation, donor age, length of pretransplant dialysis, delayed graft function, and panel reactive antibody > 30% [16].

In renal transplantation, donor age is known to have an important influence on the outcome of the graft reflecting functional renal mass [17]. In our study, higher donor age correlated with a worse graft outcome. This relationship was not found for recipient age. 

There is a long-standing debate about whether delayed graft function reduces the survival rate of a renal graft [7]. Several studies report it to be an important risk factor that influences graft survival [18, 19]. Yet, Boom et al. reported that delayed graft function did not affect the long-term survival rate of recipients they examined [20]. Our results were consistent with this finding.

Traditionally, high panel reactive antibody (PRA) has been associated with increased immunologic risk and lower kidney graft survival [21]. In our study, there was no statistically significant effect of PRA (≥30 versus <30) in pretransplant period on graft survival. 

As for HLA compatibility, it has been considered the most important independent factor for the 10-year survival rate of patients and the half-life of a patient's first renal transplant. Cecka showed 10-year survival of dead renal transplant to be of 74% when HLA was matched between the donor and recipient and stated a reduction for 58% if it was mismatched [22]. During the early posttransplant period (6 months), HLA-DR mismatches have a stronger influence on graft survival than HLA-A or B mismatches which have a very small influence. However, during the period of six months to five years posttransplantation, all three HLA loci have approximately the same influence [23]. Other studies have suggested that HLA matching is of diminishing significance, while nonimmunologic factors remain equivalently important [24]. In 2003, the US kidney allocation system was changed to eliminate priority for HLA-B similarity. Outcome analyses of the 6 years before and after the policy change found no adverse effect on graft survival [25]. Despite this, our study showed a 0 to 1 (versus 2) HLA-B mismatch to be a predictor of 10-year graft survival; HLA-A and HLA-DR mismatches had no statistical significance.

The impact of CMV serology in kidney transplantation showed donor CMV-seropositive kidneys to be associated with significantly reduced graft survival for CMV-seronegative recipients but not CMV-seropositive recipients [26]. In our study, recipient positivity for CMV IgG was statistically different between the groups and correlated with better long-term graft survival. Paired donor-recipient serology was not analyzed. 

This study presents the limitations of any retrospective study moreover this complex cohort of patients, concerning different phases of immunosuppressive regimens and the lack of accurate information of the induction and maintenance immunosuppressive strategies used; restricts more broad inferences. Nonetheless, this is a study on a large number of patients, with a long-term followup (beyond ten years posttransplant) and we could find interesting results. 

In summary, modifiable risks factors as an early KT, younger donor age, number of HLA B mismatches and an optimal first-year graft function (including absence of acute rejection episodes and creatinine level at 12 months) are of paramount importance for long-term graft survival. Measures that address these issues (careful donor selection, preemptive KT, and effective immunosuppressive protocols) are still warranted.

## Figures and Tables

**Table 1:** Recipient, donor, and kidney transplant characteristics of the enrolled population (*n* = 892).

Characteristics	Patients
Recipient	
Age (years), median [IQR]	37.0 [27.6–47.1]
Male gender, *n* (%)	530 (59.4%)
Time on dialysis (years), median [IQR]	2.6 [1.4–5.0]
Body mass index (kg/m^2^), median [IQR]	22.4 [20.3–24.2]
Dialysis technique	
Hemodialysis, *n* (%)	864 (96.3%)
Peritoneal dialysis, *n* (%)	16 (1.8%)
Prior kidney transplant, *n* (%)	73 (8.1%)
Positive CMV IgG, *n* (%)	613 (68.3%)
Prior blood transfusion (≥3 units), *n* (%)	350 (39.1%)
Hepatitis B/C+, *n* (%)	230 (25.6%)
Donor	
Age (years), median [IQR]	25.0 [18.0–39.0]
Male gender, *n* (%)	636 (70.9%)
Serum creatinine (mg/dL), median [IQR]	1.0 [0.8–1.2]
Cadaveric donor, *n* (%)	888 (99.0%)
Transplant	
0 mismatches HLA-B, *n* (%)	181 (20.2%)
1 mismatches HLA-B, *n* (%)	249 (27.8%)
2 mismatches HLA-B, *n* (%)	428 (47.7%)
PRA ≥ 30%, *n* (%)	34 (3.8%)
ATG induction protocol, *n* (%)	411 (45.8%)
Delayed graft function, *n* (%)	294 (32.8%)
Acute rejection, *n* (%)	283 (31.5%)
Serum creatinine at 12 months (mg/dL), median [IQR]	1.40 [1.20–1.70]

IQR: interquartile range; IgG CMV: immunoglobulin cytomegalovirus; HLA: human leucocyte antigen; PRA: panel reactive antibody; ATG: antithymoglobulin.

**Table 2:** Comparison of recipient, donor, and renal transplant characteristics between the two groups (graft function after 10 years versus graft loss within 10 years).

Characteristics	G I (graft function after 10 years)	G II (graft loss within 10 years)	*P* value
Recipient			
Age (years), median [IQR]	36.1 [28.1–47.1]	35.8 [26.9–47.2]	**Ns**
Male gender, *n* (%)	146 (57.5%)	384 (60.2%)	**Ns**
Time on dialysis (years), median [IQR]	2.7 [1.3–4.7]	2.5 [1.4–5.6]	**Ns**
Body mass index (kg/m^2^), median [IQR]	22.4 [20.3–24.2]	22.2 [19.9–24.2]	**Ns**
Prior kidney transplant, *n* (%)	56 (8.8%)	17 (6.7%)	**Ns**
Positive CMV IgG, *n* (%)	462 (73.9%)	151 (62.1%)	**0.001**
Prior blood transfusion (≥3 units), *n* (%)	235 (36.8%)	115 (45.3%)	**0.023**
Donor			
Age (years), median [IQR]	24.0 [18.0–36.0]	29.0 [20.0–46.0]	**<0.001**
Male gender, *n* (%)	454 (77.2%)	182 (74.6%)	**Ns**
Serum creatinine (mg/dL), median [IQR]	1.0 [0.8–1.2]	1.0 [0.8–1.2]	**Ns**
Transplant			
0 mismatches HLA-B, *n* (%)	139 (22.7%)	42 (17.0%)	**0.002**
1 mismatches HLA-B, *n* (%)	189 (30.9%)	60 (24.3%)	
2 mismatches HLA-B, *n* (%)	283 (46.3%)	145 (58.7%)	
PRA ≥ 30%, *n* (%)	18 (3.5%)	16 (7.6%)	**0.031**
ATG induction protocol, *n* (%)	313 (45.5%)	98 (39.0%)	**0.005**
Delayed graft function, *n* (%)	187 (29.3%)	107 (42.1%)	**<0.001**
Acute rejection, *n* (%)	163 (25.3%)	120 (47.2%)	**<0.001**
Serum creatinine at 12 months (mg/dL), median [IQR]	1.30 [1.10–1.60]	1.60 [1.33–2.10]	**<0.001**

IQR: interquartile range; IgG CMV: immunoglobulin cytomegalovirus; HLA: human leucocyte antigen; PRA: panel reactive antibody; ATG: antithymoglobulin.

**Table 3:** Long-term graft survival predictors; multivariate-adjusted logistic regression.

Characteristics	OR	CI	*P* value
Recipient			
Gender (male versus female)	1.84	1.21–2.80	**0.005**
Time on dialysis (months)	0.93	0.87–0.90	**0.044**
Positive CMV IgG (versus negative)	1.59	1.02–2.49	**0.040**
Donor			
Age (years)	0.98	0.97–0.99	**0.004**
Transplant			
0-1 HLA-B mismatch (versus 2)	1.80	0.28–0.84	**0.004**
Absence of acute rejection (versus presence)	0.64	0.41–0.99	**0.047**
Serum creatinine 12 months after (mg/dL)	0.26	0.16–0.41	**<0.001**

Variables included in the multivariable model: age and sex of the recipient, mean time on dialysis, recipient status for cytomegalovirus IgG, prior blood transfusions (<3 versus ≥3), age of the donor, number of HLA-B mismatches (0, 1 versus 2), PRA (<30 versus ≥30%), induction with antithymoglobulin, delayed graft function, acute rejection, and serum creatinine at 12 months after KT.
